# Determination of Ultrastructural Properties of Human Carotid Atherosclerotic Plaques by Scanning Acoustic Microscopy, Micro-Computer Tomography, Scanning Electron Microscopy and Energy Dispersive X-Ray Spectroscopy

**DOI:** 10.1038/s41598-018-37480-z

**Published:** 2019-01-24

**Authors:** Bukem Bilen, Leyla Turker Sener, Isil Albeniz, Meltem Sezen, Mehmet Burcin Unlu, Murat Ugurlucan

**Affiliations:** 1 Bogazici University, Department of Physics, Istanbul, 34342 Turkey; 2 Istanbul University, Istanbul Faculty of Medicine, Biophysics Department, Istanbul, 34093 Turkey; 3 Sabanci University, Nanotechnology Research and Application Center, Istanbul, 34956 Turkey; 4 Istanbul University, Istanbul Faculty of Medicine, Cardiovascular Surgery Department, Istanbul, 34093 Turkey

## Abstract

Microcalcification is the precursor of vulnerability of plaques in humans. Visualization of such small structures *in vivo* with high spatial resolution is an unsolved issue. The goal of this study is to evaluate the potential of scanning acoustic microscopy (SAM) in the determination of atherosclerotic plaques with calcifications by validating this technique with micro-computer tomography (micro-CT), scanning electron microscopy (SEM) and energy dispersive X-ray spectroscopy (EDS). The fibrocalcific plaques were obtained from 12 different patients and initially examined with micro-CT. The images exhibited calcifications within these plaques. For imaging with SAM, approximately 5 *μ*m thick slices were prepared. Sound speed values within calcified regions were measured to be greater than the ones in collagen-rich regions. These fibrocalcific plaques were also examined with SEM and EDS revealing collagen and calcium deposition within these samples. The consistency of the results obtained by all of the modalities involved in our study is an indication of the potential of SAM as a clinical tool for the diagnosis of vulnerable plaques.

## Introduction

Microcalcifications or spotty calcifications within the plaques are the indicators of plaque vulnerability^1,2^, where, larger calcifications are found to be stable and no longer threatening^3,4^. Micro-CT is an accurate imaging modality for the detection of micrometer-sized structures, which provides much higher resolution than cone beam computed tomography (CBCT) does. Micro-CT generally has a resolution of less than 10 *μ*m voxel size, while, the resolution of CBCT ranges between 76 *μ*m to 400 *μ*m^5–7^. Micro-CT can perform *in-vitro* processing of the structure of materials such as composites, polymers, biological materials (bone, teeth, cartilage tissue) and imaging of up to 4 different substances in a material^8^ using X-rays. Sample preparation and positioning is simple and does not require high vacuum or low temperatures that may adversely affect the structure. On the other hand, micro-CT is an expensive diagnostic imaging tool and also requires an ionizing radiation, therefore, seeking an alternative is inevitable.

Scanning electron microscopy (SEM) produces images with a resolution in the order of a nanometer, by scanning the surface of the specimen with a focused beam of electrons. Energy dispersive X-ray spectroscopy (EDS) is a chemical characterization technique, which detects all elements ranging from beryllium (Be) to uranium (U) and their distribution within samples, also by the bombardment of the specimen surface with a focused electron beam. EDS can be implemented in electron microscope systems and consequently, both morphological and chemical information about the sample can be obtained. Microcalcification analysis can easily be performed with SEM-EDS; however, this would again be a very expensive method.

Scanning acoustic microscopy (SAM), which is a non-invasive and comparably less expensive technique, provides information about the morphology and the mechanical properties of the specimen simultaneously at microscopic levels, rapidly and without a need for special staining. Focused high frequency ultrasound signals are used to calculate either the speed of sound (SOS) through samples^9–18^ or acoustic impedance of samples^19,20^. Two-dimensional maps of an area of around 5 × 5 mm are obtained in a couple of minutes. Similarly, cells and organelles can be examined using higher frequencies of 100 to 1200 MHz^21–28^.

In our study, we evaluated the ultrastructural properties of the human carotid plaques with scanning acoustic microscopy, micro-computer tomography, scanning electron microscopy and energy dispersive X-ray spectroscopy. The consistency of results obtained by SAM, SEM, EDS and micro-CT is an evidence of the potential of SAM as a diagnostic tool for the determination of microcalcifications in clinics. SAM provided both structural and mechanical information with micrometer resolution about the fibrocalcific plaques and was successful in differentiating the collagen-rich areas from calcified regions. Micro-CT and SEM monitored calcifications, while, EDS provided elemental distribution within samples. Consequently, we can state that SAM is capable of identifying calcifications within the human carotid fibrocalcific plaques, which are signs of plaque vulnerability at the microscopic level and therefore, is a promising tool for the determination of life-threatening plaques in clinics.

## Results

### Micro-CT Results

**Figure 1:**
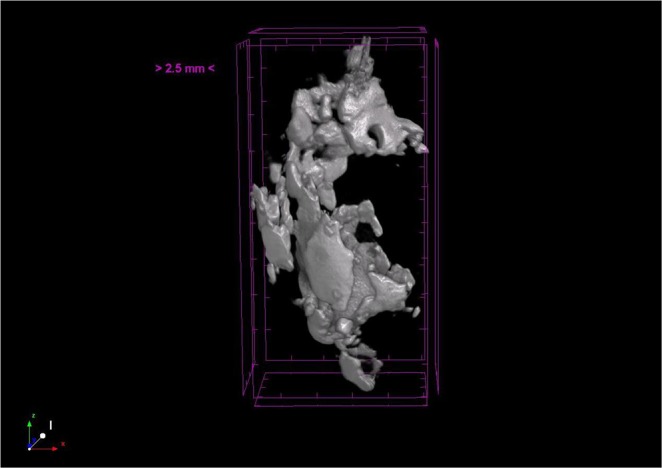
Micro-CT image of a fibrocalcific plaque sample in which the calcifications were monitored clearly in 3-dimensions.

As can be seen from Fig. 1, each plaque, fixed with gauze inside a tube full of 2% formaldehyde, was first monitored with micro-CT, to visualize calcifications spread through. The calcific regions were specified and samples containing calcifications were prepared for SAM studies.

### Scanning Acoustic Microscopy Results

**Figure 2:**
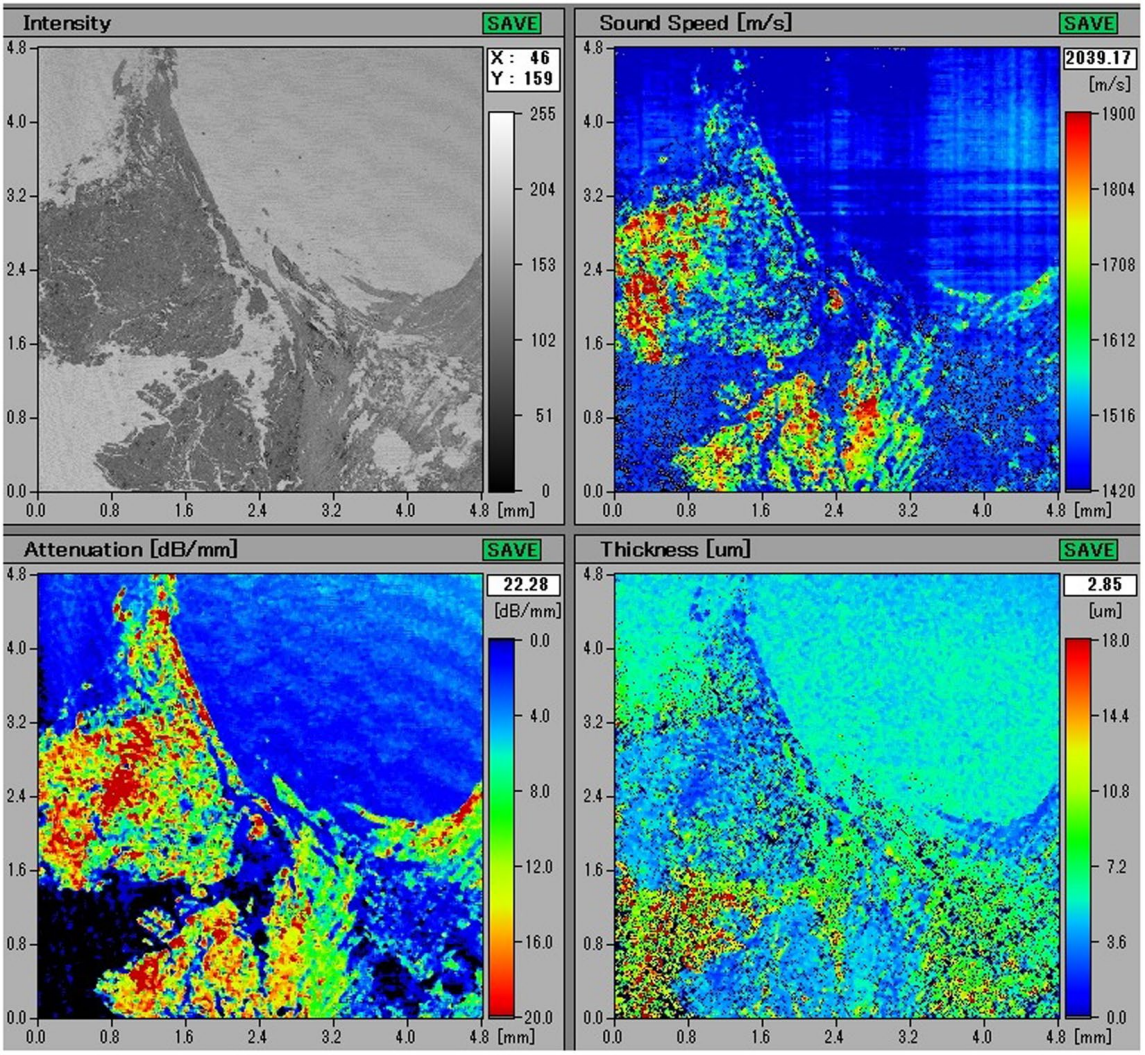
SAM images of a fibrocalcific plaque sample. Top left image is the intensity image. Top right image is the sound speed map of the sample. Bottom left image is the attenuation map within the plaque. Bottom right is the thickness map of the sample, indicating an average thickness of 5 *μ*m.

The fibrocalcific plaques, received from patients undergoing carotid endarterectomy operation, were sliced at approximately 5 *μ*m thick with a microtome and mounted on glass slides for SAM studies. The images obtained using the sound speed mode of SAM were constructed by comparing the reflections of ultrasound signals from front and rear surfaces of the slices. Figure 2 shows the maps of sound speed, attenuation and thickness distribution of a representative plaque sample. Top left image is the acoustic intensity image. Top right image is the sound speed map of the specimen, showing higher sound speed values within calcified regions than the ones within collagen-rich areas. Bottom left image shows the attenuation within the sample, which is described as the intensity divided by the thickness. Bottom right image is the thickness distribution within the slice, indicating an average thickness of 5 *μ*m.

Table 1 presents sound speed values within all the plaques examined. Fibrocalcific samples consist of mainly calcific and collagen-rich areas and calcification is easily distinguishable, since, it is quite stiff with respect to collagen. Collagen-rich and calcific regions have distinctive sound speed values due to the discrepancy in elastic properties of collagen and calcification. Each value given in Table 1 is the average obtained from multiple points within each area.

**Table 1:** Sound speed values of fibrocalcific plaques of 12 different patients with two distinguished regions of collagen and calcification.

Patients	Sound Speed (m/s) (collagen-rich region)	Sound Speed (m/s) (calcific region)
1	1680.98 ± 26.38	2016.49 ± 59.43
2	1688.63 ± 35.31	2030.49 ± 77.08
3	1629.54 ± 20.61	1839.89 ± 37.68
4	1777.64 ± 14.20	2036.55 ± 70.55
5	1658.82 ± 31.56	1841.84 ± 44.45
6	1729.55 ± 20.38	2047.21 ± 80.59
7	1757.01 ± 26.94	2030.46 ± 79.01
8	1647.60 ± 25.94	2054.40 ± 67.14
9	1708.09 ± 32.87	2002.84 ± 84.79
10	1682.09 ± 29.13	1953.72 ± 46.36
11	1669.74 ± 21.98	1993.24 ± 19.28
12	1679.89 ± 41.78	2018.31 ± 73.88

### Scanning Electron Microscopy and Energy Dispersive X-Ray Spectroscopy Results

**Figure 3:**
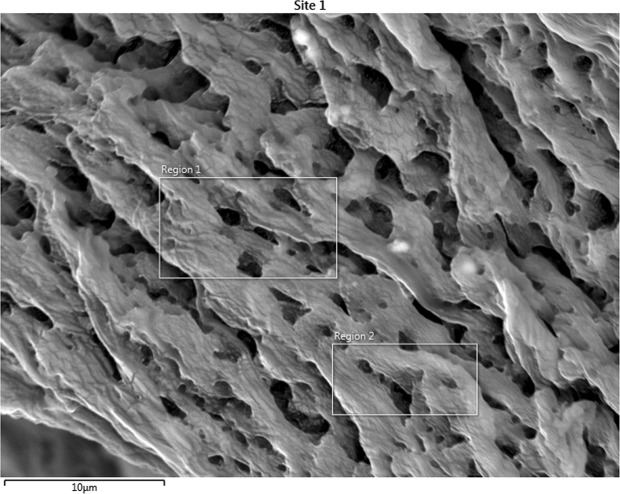
Scanning electron microscopy image of the collagen-rich region of the plaque. Energy dispersive X-ray spectroscopy was performed on the designated regions, 1 and 2.

**Figure 4:**
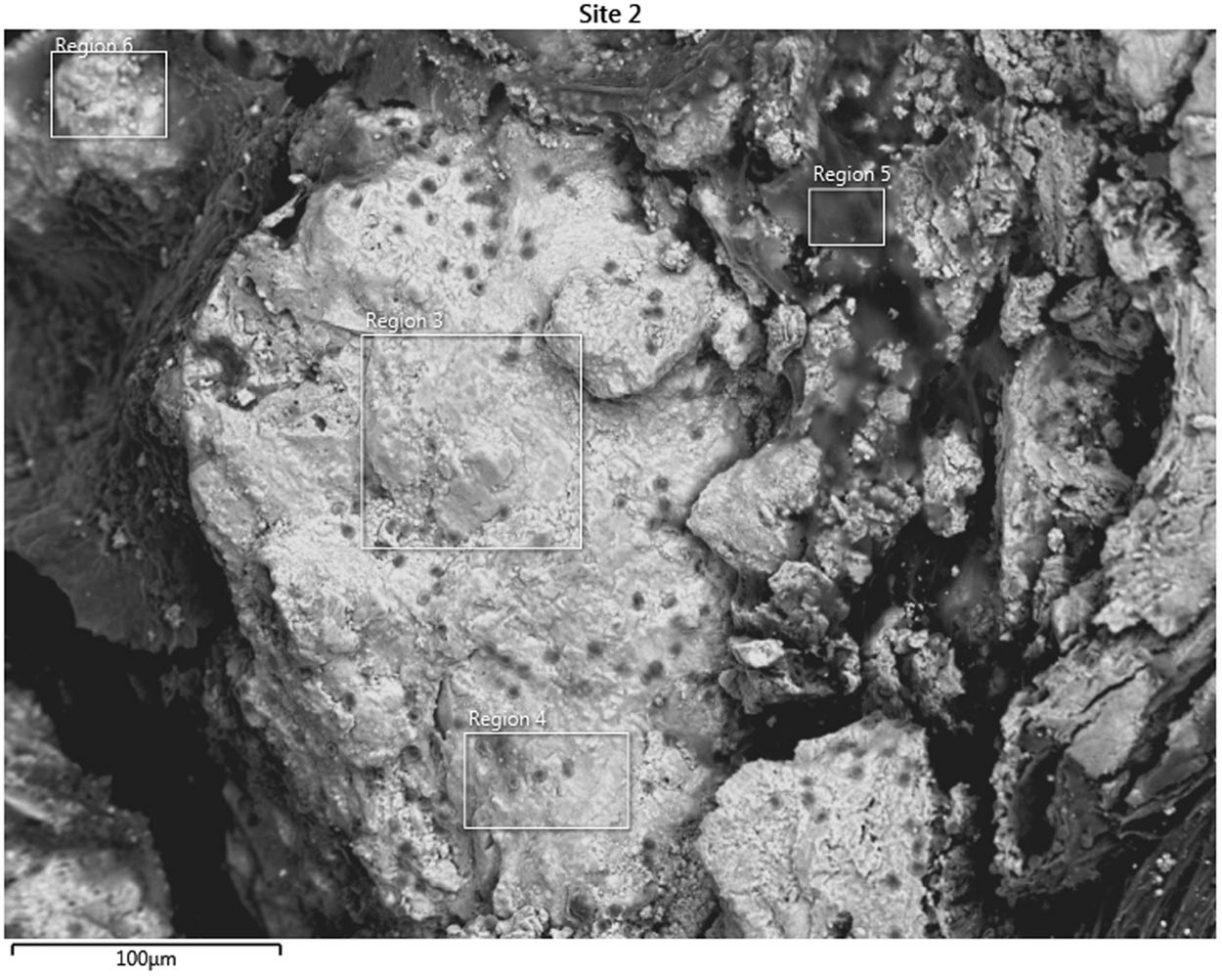
Scanning electron microscopy image of the highly calcified region of the plaque. Energy dispersive X-ray spectroscopy was performed on the designated regions, 3, 4, 5 and 6.

**Figure 5:**
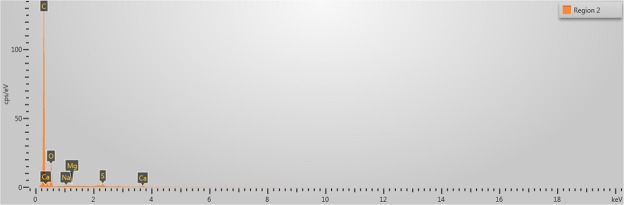
Energy dispersive X-ray spectroscopy result of the collagen rich region 2, shown in Fig. 3.

**Figure 6:**
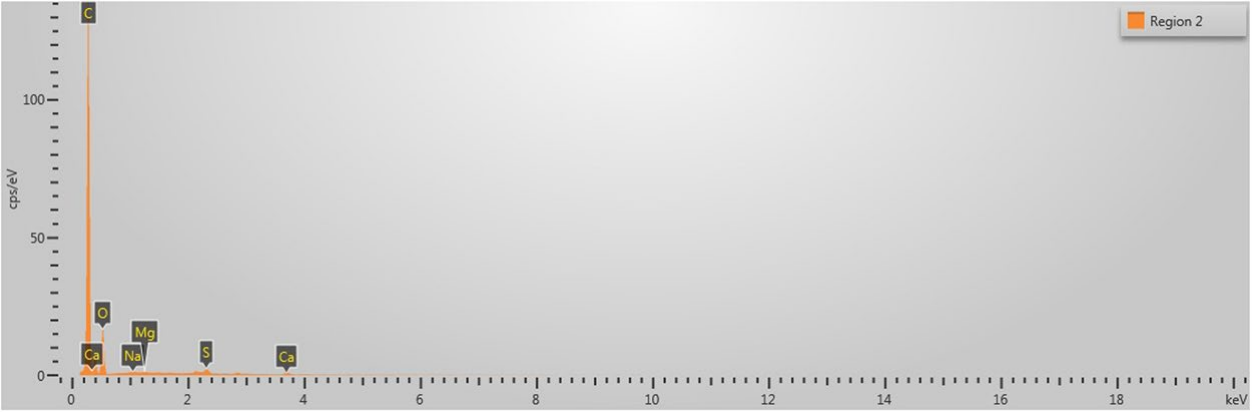
Energy dispersive X-ray spectroscopy result of the highly calcified region 3, shown in Fig. 4.

**Figure 7:**
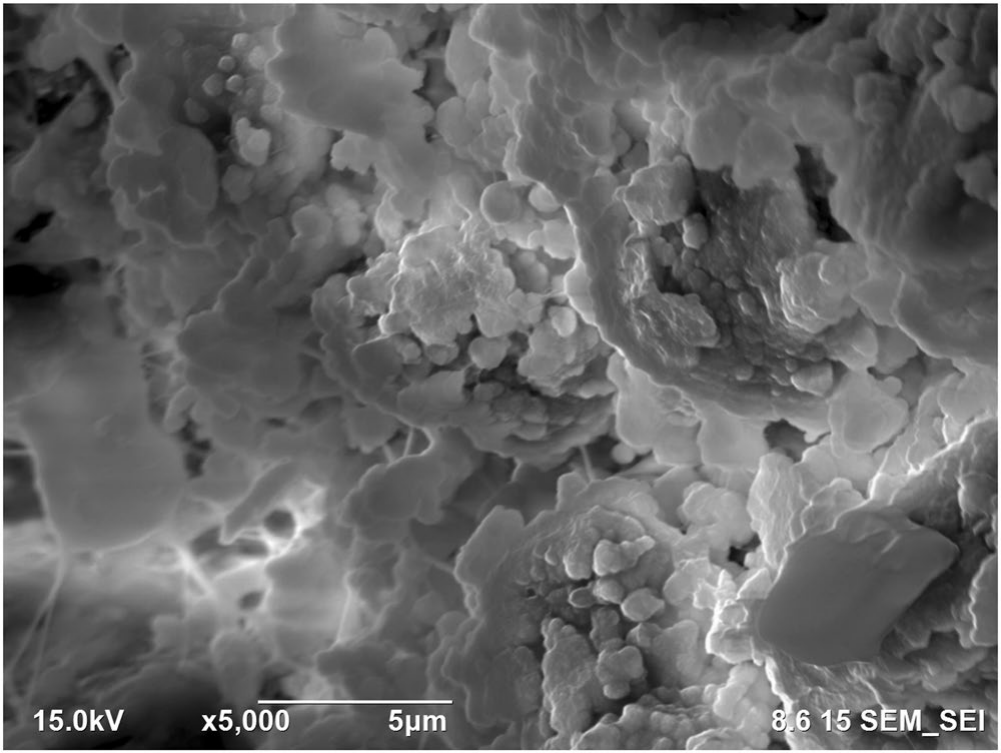
Scanning electron microscopy image of a highly calcified region with 5000x magnification. Closer look to the calcified region demonstrates calcium deposition more clearly.

SEM images of the collagen-rich and calcified regions of the plaques were obtained as shown in Fig. 3 and in Fig. 4, respectively. In Fig. 3, two regions were designated for EDS analysis. Similarly, in Fig. 4, 4 regions were specified. EDS analyses of representative regions 2 and 3 are shown in Fig. 5 and in Fig. 6, respectively. EDS results of the regions in each figure (Figs 3 and 4) were similar. SEM image of a highly calcified region with very high magnification of 5000x is also shown in Fig. 7 for a closer look to calcifications.

### Statistical Analysis of Carotid Plaques

Table 1 presents sound speed values (mean standard deviation, m/s) in collagen-rich and calcific regions. In calcified regions significantly higher sound speed values were obtained. Statistical differences between these two regions were determined and the level of statistical significance was set to p < 0.01.

## Discussion

Carotid endarterectomy is still the gold standard treatment procedure for atherosclerotic carotid artery disease^29–32^. However, there is a controversy regarding the management of asymptomatic patients, whether to operate, stent or medically follow these cases. There are worldwide many clinics accepting patients, surgical or interventional candidates with high grade carotid artery stenosis, whereas, various others enrolling only symptomatic patients with important carotid artery stenosis. In the recent guidelines carotid endarterectomy or stenting is proposed in patients with high grade carotid stenosis with low periprocedural risks as a class II indication with a level of evidence of B^33^. In order to be more selective to intervene on asymptomatic patients with carotid stenosis, literature lacks established criteria indicating which patients are prone to stroke in the early or long run. There are studies researching the plaque features^34^ and various others for prediction of cerebrobascular events^35^, however, none could be 100% reliable so far. Histopathological structure of atheromatous plaques including carotid plaques has been widely studied. There is sparse information regarding the ultrastructural properties, chemical composition and mineral component of these materials^36^. A study on lipid-rich plaques of human abdominal aorta was conducted using photoacoustic imaging and the feasibility of visualizing lipid-rich plaques at wavelengths of 970 and 1210 nm was tested^37^. The photoacoustic spectra obtained in a region within the plaque showed the spectral signature of lipids. In addition, the lipid-rich plaque was imaged successfully, while illuminating the sample through 2.8 mm of blood, demonstrating the possibility of implementing the photoacoustic technique *in vivo*. In our study, fibrocalcific human carotid plaques were examined in detail with scanning acoustic microscopy (SAM), micro-computer tomography (micro-CT), scanning electron microscopy (SEM) and energy dispersive X-ray spectroscopy (EDS). All of the plaques were fibrocalcific with an insignificant lipid content, therefore, we observed only collagen-rich and calcific regions. As can be seen in Fig. 1, calcification is pronounced in a fibrocalcific plaque. The regions containing calcifications were chosen for observation with SAM. SAM in sound speed mode was capable of differentiating morphological and mechanical properties of collagen-rich and calcified regions of the fibrocalcific plaque samples. Sound speed values of calcifications in all samples were higher than the ones of collagen-rich regions, which is in agreement with literature^10,12^. Calcific regions were easily distinguishable, since they were dark-colored, in fact, in some samples they were black due to former harmless thrombosis. The calcified regions of the fibrocalcific plaques were stiff with higher elastic moduli, therefore, sound speed values were calculated to be greater than the ones of the collagen-rich regions. The difference in sound speed values of calcific regions in all samples was due to the difference in calcification amounts, which vary a lot from one patient to the other, even the symptoms are the same. The patients had undergone surgeries after either they experienced a cerebrovascular incident or had 70% and above shrinkage in their internal carotid artery (ICA), without any further information about the history of the pathology of the plaques and their progression. Besides, some patients are asymptomatic and they have been diagnosed by chance. Two dimensional maps of sound speed with a micrometer resolution, as in Fig. 2, make SAM a suitable tool for the determination of microcalcifications, which are indicators of vulnerability. Microcalcifications in other tissue types can also be examined by SAM, since calcium deposition is stiffer than any human tissue with a greater acoustic impedance value^38,39^, and therefore easily distinguishable within tissues like breast or prostate. Doppler ultrasonography has been widely used to characterize carotid plaques by obtaining plaque morphology, which is an indicator of vulnerability^40^. SAM, on the other hand, can provide morphological and chemical information about the plaques simultaneously, which enables diagnosis of vulnerable plaques with a verification.

SEM images were used to differentiate collagen-rich and calcific regions in fibrocalcific plaques and EDS analyses were performed for determining the compositional differences between two regions of interest. In Fig. 3, we observed collagen-rich region in one plaque sample and two regions were chosen for EDS analysis. Similarly, in Fig. 4, calcification-rich region was visualized in one sample and four regions were chosen for EDS analysis. These chosen regions on each figure gave similar EDS results. As can be seen in Fig. 5, in collagen-rich regions, calcium deposition is insignificant, while, in calcified regions, as shown in Fig. 6, calcium peak is pronounced. Calcium (Ca), oxygen (O) and phosphate (P) elements observed in the SEM-EDS analysis, might be in the form of hydroxyapatite and calcium oxalate phases. However, EDS analysis was used to only determine the elemental composition distribution and the accumulation of calcium in particular regions. A closer look into a calcified region in Fig. 7 demonstrated calcium deposition more clearly. In another study on carotid plaques, highly abundant phosphorus and calcium, forming calcium phosphates and reduced levels of sodium, were determined with SEM-EDS^36^. In addition, the X-ray diffraction yielded three groups of materials such as predominant hydroxyapatite-type crystals, crystalline materials containing amorphous component and as a last subtype, wholly amorphous material. The most abundant mineral in their atheromatous plaques, as in our materials has been hydroxyapatite. The hydroxyapatite contained crystals of cholesterol and lipid nuclei in different layers, which was a reflection of plaque to be in different stages of its formation. Depending on the SEM-EDS results of our study, the volume difference of calcium and sodium concentrations in arteries with and without atheromatous plaques may be an indicator of relationship in pathologic development of calcium deposits.

In this study, we tried to evaluate the potential of SAM in the characterization of fibrocalcific plaques by validating its outcomes with the results of SEM, EDS and micro-CT. The atherosclerotic plaques, before becoming fibrocalcific, are vulnerable and therefore, had a high risk to rupture. Fibrocalcific plaques consist of mainly collagen and calcifications and are said to be stable, however, at the formation stage of microcalcifications, fibrous cap, covering a lipid-rich core, becomes thin and weakened, making the plaque vulnerable. We observed various elemental compositions in forms of hydroxyapatite and calcium oxalate in the extracted calcium plaques of the patients. Therefore, imaging of calcifications at micrometer scale is very substantial. Sound speed maps of the fibrocalcific samples show clearly different values in collagen-rich and calcified regions. Consequently, monitoring capacity of SAM with micrometer resolution strengthen the idea of introducing SAM as an imaging tool for vulnerable plaques.

## Materials and Methods

This study was approved by Ethics Committee of Istanbul University Medical Faculty (No: 2018/952). All research was performed in accordance with relevant guidelines/regulations.

### Surgical Technique and Sampling

Patients were enrolled to the study after being informed about the research and following their consent to donate the left minimum amount of blood from the blood drawn to perform routine preparatory laboratory analysis and their carotid plaques, which are collected and histopathologically examined when they were presumed unusual as an institutional health policy. The surgical procedures and sampling were performed by the same surgeon (M.U.) in all the patients using the same standard technique. Blood samples were drawn (5 cc) into dry tube and (2 cc) into ethylenediaminetetraacetic acid (EDTA) tube after insertion of venous line and invasive artery catheter. The operations were performed with deep and/or superficial regional anesthesia. Superficial cervical block was performed with the injection of 10 cc bupivacaine of 0.05%, 6 cc lidocaine of 2% and 4 cc saline combination along the lateral border of the sternocleidomastoid muscle subcutaneously. Deep cervical plexus block was performed at the level of the transverse processes of cervical vertebrae C2, C3, and C4. A combination of lidocaine hydrochloride and bupivacaine hydrochloride was injected after negative aspiration result for blood. A total amount of bupivacaine hydrochloride of 2–3 mg/kg was allowed. Additional prilocaine hydrochloride was used subcutaneously at the incision line as infiltration anesthesia. Additional doses of prilocaine hydrochloride was injected intraoperatively if the patient complained of pain during the procedure. The allowed total dose of prilocaine was 5 mg/kg. Intraoperative remifentanil (0.025–0.05 mg/kg/min) maintained an adequate level of comfort, responsiveness, and cooperation. Continuous infusion of nitroglycerin was used for blood pressure control. Additional diltiazem or metoprolol was administered if needed. After de-clamping, occasionally midazolam was given. Systemic heparin (100 IU/kg) was injected before clamping and was not antagonized after the procedure. A shunt was selectively used in case of neurologic deterioration at cross-clamping test with a duration of 2–3 minutes. A standard incision parallel to the sternocleidomastoid muscle was performed and common carotid artery (CCA), internal carotid artery (ICA) and external carotid artery (ECA) were prepared and dissected. After systemic heparin injection, the arteries were clamped. The consciousness and the neurologic status of the patient were evaluated with patient’s response to verbal stimuli and ability to move contralateral side hand and foot for at least 2–3 minutes prior to arteriotomy. In case of neurologic disturbance, endarterectomy was performed with insertion of a shunt. A longitudinal incision from the common carotid artery to the disease-free level of internal carotid artery was performed. Thromboendarterectomy was performed using an elevator. The excised atherosclerotic plaque was embedded into 2% formaldehyde for experimental investigation. The arteries were reconstructed with patch in all patients. Following hemostasis, a drain was placed to the surgical site and tissues were sutured anatomically. The symptomatic otherwise more stenotic side was prioritized followed by the surgery of the contralateral side, in cases with bilateral carotid disease.

### Micro-Computer Tomography

Plaques fixed within 2% formaldehyde were scanned using a Skyscan 1174v2 device (Bruker, Kontich, Belgium) with the following settings: 50 kV, 800 *μ*A, 1024 × 1304 resolution, filter of 0.5 mm aluminum, rotation step of 0.9°, and 40 W power. After obtaining micro-focal spot and arranging high-resolution detectors for X-rays, full-scan mode 360° was performed for each plaque in about 50 min scan period and images were created via Ctvox and ctan programs. Reconstruction of 480 raw images in TIFF format was performed using NRecon Software (Bruker, Kontich, Belgium) generating 755 horizontal sections in BMP format.

### Scanning Acoustic Microscopy

**Figure 8:**
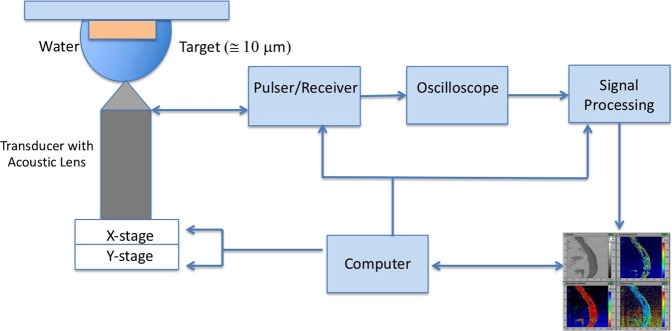
SAM setup. SAM has a transducer with quartz lens, a pulser/receiver, an oscilloscope, a stage control computer and a display monitor.

Approximately 5 *μ*m thick samples were characterized by scanning acoustic microscope (AMS-50SI), developed by Honda Electronics (Toyohashi, Japan). Figure 8 shows the SAM setup in sound speed mode. SAM has a transducer with quartz lens, a pulser/receiver, an oscilloscope, a stage control computer and a display monitor. An 80 MHz transducer, which has a spot size of 17 *μ*m and a focal length of 1.5 mm, generates single pulses of width of 5 ns with a repetition rate of 10 kHz and also collects the acoustic waves. Water is the coupling medium between the quartz lens and the target. X-Y stage, controlled by a computer, scanned the transducer and therefore the target material. The reflected signals from both front and rear sides of the target were collected by the 80 MHz transducer and analyzed by the oscilloscope. Finally, intensity, sound speed, attenuation and thickness maps of the region of interest with 300 × 300 sampling points were obtained with a lateral resolution of approximately 20 *μ*m.

**Figure 9:**
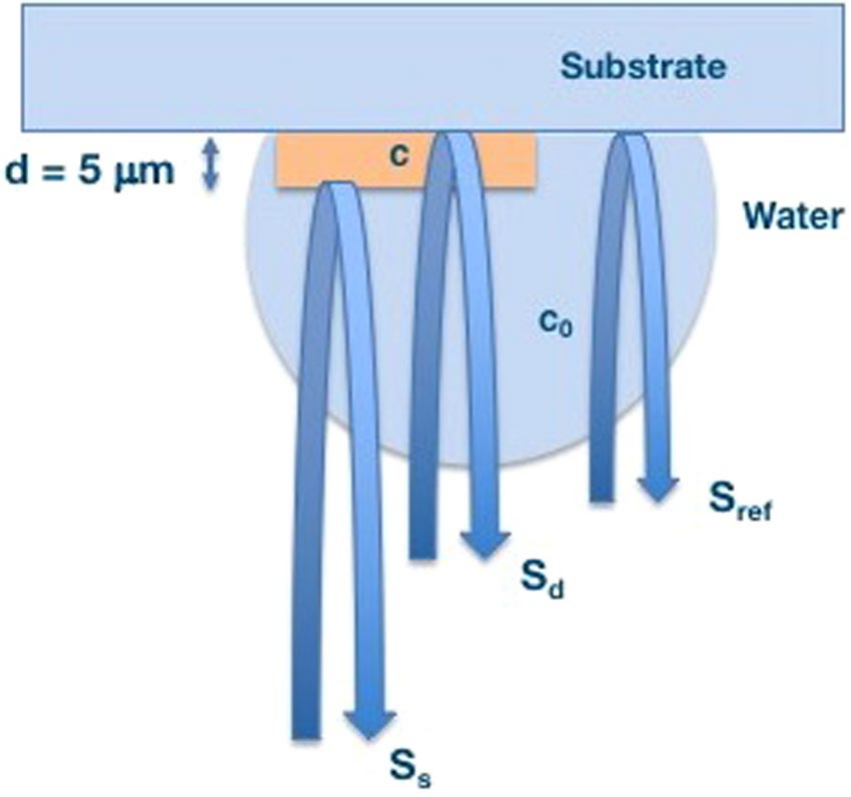
Principle of SAM in sound speed mode. Reflections from front and rear sides of the tissue are compared to measure the thickness of the tissue, sound speed and attenuation within the sample. *S*_*s*_ is the signal reflected from the rear surface of the target, *S*_*d*_ is the signal reflected from the rear surface of the target and *S*_*ref*_ is the reflected signal from the substrate.

Figure 9 shows the principle of SAM in sound speed mode. The target is the 5 *μ*m thick plaque sample placed on a flat glass slide. The signal generated by the transducer is transmitted through the coupling medium and focused on the target. *S*_*ref*_ is the reflected signal from the glass surface without the target. The reflected signal from the target received by the same transducer is composed of the interference of reflection from front (*S*_*s*_) and rear (*S*_*d*_) surfaces of the tissue slice, *S*_*s*_ + *S*_*d*_. These two components can not be separated in time domain and therefore, intensity and phase spectra are determined by Fourier transformation into frequency domain. The phase of the reflection wave from front surface, *ϕ*_*front*_, and the phase of the reflection wave from rear surface, *ϕ*_*rear*_ can be written as^41^

1 $$2\pi f\times \frac{2d}{{c}_{0}}={\varphi }_{front}$$

2 $$2\pi f\times 2d(\frac{1}{{c}_{0}}-\frac{1}{c})={\varphi }_{rear}$$

where *f* is the frequency of the signal, *d* is the thickness of the tissue slice, *c*_0_ is the sound speed within water and *c* is the sound speed within the tissue slice. Thickness of the target can be written as

3 $$d=\frac{{c}_{0}}{4\pi f}{\varphi }_{front}$$

Sound speed can be written as

4 $$c={(\frac{1}{{c}_{0}}-\frac{{\varphi }_{rear}}{4\pi fd})}^{-1}$$

Attenuation of ultrasound signal within the tissue can be calculated by dividing the amplitude by thickness.

### Scanning Electron Microscopy and Energy Dispersive X-ray Spectroscopy

Scanning electron microscopy and energy dispersive X-ray spectroscopy studies were performed in JEOL JIB 4601 Focused Ion Beam Scanning Electron Microscope multi-beam platform coupled with Oxford X-maxN Energy Dispersive X-Ray Spectroscopy system.

### Statistical Analysis

Using SPSS for Windows version 19.0 (SPSS, Chicago, IL, USA), all statistical analyses were performed. Mean, standard deviation, rate and frequency values were used for the descriptive statistics of the data. The evaluation of the distribution of continuous variables was done by Kolmogorov-Smirnov test. Student’s t-test and Mann-Whitney U test were used for the analysis of parametric and non-parametric data, respectively. Comparison of the categorical variables between groups was done by *χ*^2^ test. Pearson correlation analysis was used for the correlation analysis and logistic regression analysis was used for the determination of the impact of variables. Standardized *β* coefficients and 95% confidence intervals (CI) were calculated. Statistical significance was defined as p < 0.01.

### Limitations

The small number of the samples is the major limitation of the study. However, similar results obtained with different detailed high technology imaging equipments within the 12 samples has been accepted as sufficient to reach out a conclusion regarding the ultrastructural composition of atheromatous carotid plaques. Another limitation may be accepted as the unavailability of correlation data of plaques in symptomatic and asymptomatic patients, however, a correlation study will be conducted in a larger cohort and presented later on.

## Data Availability

Corresponding author can provide the datasets of this study on reasonable request.
